# The Association Between Internet Use and Ambulatory Care-Seeking Behaviors in Taiwan: A Cross-Sectional Study

**DOI:** 10.2196/jmir.5498

**Published:** 2016-12-07

**Authors:** Ronan Wenhan Hsieh, Likwang Chen, Tsung-Fu Chen, Jyh-Chong Liang, Tzu-Bin Lin, Yen-Yuan Chen, Chin-Chung Tsai

**Affiliations:** ^1^ Department of Medicine National Taiwan University College of Medicine Taipei Taiwan; ^2^ Institute of Population Health Sciences National Health Research Institutes Miao-Li Taiwan; ^3^ National Taiwan University College of Law Taipei Taiwan; ^4^ Graduate Institute of Digital Learning and Education National Taiwan University of Science and Technology Taipei Taiwan; ^5^ Department of Education National Taiwan Normal University Taipei Taiwan; ^6^ Graduate Institute of Medical Education & Bioethics National Taiwan University College of Medicine Taipei Taiwan

**Keywords:** mass media, Internet, literacy, outpatient clinic, ambulatory care

## Abstract

**Background:**

Compared with the traditional ways of gaining health-related information from newspapers, magazines, radio, and television, the Internet is inexpensive, accessible, and conveys diverse opinions. Several studies on how increasing Internet use affected outpatient clinic visits were inconclusive.

**Objective:**

The objective of this study was to examine the role of Internet use on ambulatory care-seeking behaviors as indicated by the number of outpatient clinic visits after adjusting for confounding variables.

**Methods:**

We conducted this study using a sample randomly selected from the general population in Taiwan. To handle the missing data, we built a multivariate logistic regression model for propensity score matching using age and sex as the independent variables. The questionnaires with no missing data were then included in a multivariate linear regression model for examining the association between Internet use and outpatient clinic visits.

**Results:**

We included a sample of 293 participants who answered the questionnaire with no missing data in the multivariate linear regression model. We found that Internet use was significantly associated with more outpatient clinic visits (*P*=.04). The participants with chronic diseases tended to make more outpatient clinic visits (*P*<.01).

**Conclusions:**

The inconsistent quality of health-related information obtained from the Internet may be associated with patients’ increasing need for interpreting and discussing the information with health care professionals, thus resulting in an increasing number of outpatient clinic visits. In addition, the media literacy of Web-based health-related information seekers may also affect their ambulatory care-seeking behaviors, such as outpatient clinic visits.

## Introduction

The way people search for health information is constantly changing. Not long ago, people relied almost solely on physicians’ advice to address patients’ medical needs because medicine was a highly specialized field of knowledge, inaccessible and incomprehensible to the general population. However, recent studies have indicated that the picture is gradually changing [1].

According to an annual report published by the Taiwan Network Information Center in 2012, 15.94 million Taiwanese used the Internet, accounting for 77.25% of the Taiwanese population aged 12 years and older [2]. Among those Internet users, many had used the Internet for searching health-related information. Research reported that, in the United States in 2009, 74% of adults used the Internet, and 61% had searched for health-related information online. In particular, 49% had accessed a website for understanding a specific medical condition or problem [3]. As reported by the Pew Research Center in 2013, approximately 59% of Americans had searched online health information in the preceding year [4]. The Internet offers a wide variety of health-related information, allowing the general population to reaffirm what they have heard from their doctors and to make a thoroughly informed decision [5-7].

Compared with the traditional ways of gaining health-related information, such as visiting an outpatient clinic, reading newspapers or magazines, listening to radio or watching television shows, getting advice from neighbors or community or family members, the Internet is inexpensive, accessible, and conveys diverse opinions. On the other hand, more and more physicians use the convenience of the Internet to search drug information [8] and Web-based evidence sources such as UpToDate [9] for providing better medical care to patients.

Relevant studies on how increasing Internet use affects outpatient clinic visits have been inconclusive. Azocar et al [10] in 2003 concluded that patients’ use of a behavioral health website significantly motivated them to increase their use of health care services. In 2008, Lee [11] reported that increasing use of the Internet was correlated with increasing outpatient clinic visits, although only age and sex were controlled in the data analysis. Another study analyzed the association between Internet use and the Internet users’ health-seeking behaviors for those who searched the Web for different purposes. The authors reported that, for every 1-hour increment of searching the Internet, the likelihood for patients to visit physicians was increased by about 10% [12].

In contrast, some studies found a negative or no effect of using the Internet on the number of outpatient clinic visits. A survey conducted in the United States showed that 94% of the participants said their Internet use did not change the number of outpatient clinic visits they made [13]. Another survey conducted in Japan reported that 88.9% of the respondents thought there was no association between their Internet use and their frequency of visiting outpatient clinics or making phone calls to their physicians to inquire about health-related issues [14].

Given that the association between Internet use and health-seeking behaviors is still controversial, and that prior studies were conducted without sufficiently adjusting for potential confounding variables, we conducted this survey in which we derived the data from a sample randomly selected from the general population in Taiwan, with adjustment for most of the potential confounding variables. The aim of this study was to examine the role of Internet use in health-seeking behaviors as indicated by the number of outpatient clinic visits.

## Methods

We derived the data for this study from the sixth cycle’s second year (2011) survey of the research project Taiwan Social Change Survey (TSCS) [15]. The project was conducted by the Institute of Sociology, Academia Sinica, and sponsored by the Ministry of Science and Technology (formerly known as the National Science Council) in Taiwan. Each year, 2 modules were used for the questionnaires. For example, in 2009, the 2 modules were Social Inequality and Religion. A total of 2026 Social Inequality questionnaires and 1924 Religion questionnaires were finally answered by the participants randomly selected from Taiwan’s general population.

The 2011 TSCS survey contained 2 modules: (1) the Family questionnaire, and (2) the Health questionnaire. Participants in the 2011 TSCS survey were randomly selected from the general population in Taiwan. Face-to-face structured interviews were conducted for all selected participants. In addition to shared questions, some of the survey questions in the questionnaire had been separated into 2 sets: set-A questions and set-B questions. Each set-A question has a similar set-B question. For example, question D1a1 in set A and question D1b1 in set B both inquire about participants’ smoking status.

This was a cross-sectional study. The Health questionnaires collected for this study were answered by the participants who were randomly selected from Taiwan’s general population. Participants in this study were numbered: the odd-numbered participants were assigned to answer set-A questions and the shared questions; and the even-numbered participants were assigned to answer set-B questions and the shared questions in the Health questionnaires. Our study used a secondary dataset collected in 2011 by the Institute of Sociology in Academia Sinica.

We selected the following variables: (1) reported confounding variables such as age [16,17], sex [13,16], annual income [16], educational level [14], self-reported health status, and chronic disease [17,18]; (2) background information of a participant such as residence and marital status; (3) social support variables such as the total number of family members and self-perceived neighbor support; and (4) attitudes toward the health care system.

For balancing the variables between the group of questionnaires without missing data and the group of questionnaires with missing data, we built a model for propensity score matching using multivariate logistic regression. Age and sex were the independent variables, and whether a questionnaire answered by a participant had missing data (the uncompleted group) or not (the completed group) was the dependent variable in the multivariate logistic regression model for propensity score matching. We obtained each participant’s propensity score of being assigned to the completed group based on the multivariate logistic regression model. A participant in the completed group was matched to a participant in the uncompleted group using 1-to-1 nearest-neighbor matching without replacement and a caliper of 0.18 of the pooled standard deviation of the logit of the propensity scores. We examined whether the propensity score model had good discrimination by using the area under the receiver operating characteristic curve. We expected age and sex in the propensity score model to be balanced between the completed and uncompleted groups. The questionnaires in the completed group that were matched to the questionnaires in the uncompleted group were retained for further analysis.

For examining the linear association between an independent variable and the outcome variable, we calculated the Pearson correlation coefficient or Spearman rank correlation coefficient depending on the scale of an independent variable. The outpatient clinic visit was the outcome variable. We coded the outpatient clinic visit using a Likert scale ranging from 1 to 5, indicating the frequency of outpatient clinic visits from “never” to “several times a month,” respectively. An independent variable with a *P* value of the correlation coefficient <.30 was eligible to enter the multivariate linear regression model. In addition, we examined the collinearity between 2 independent variables using the Spearman rank correlation coefficient: “How is your health status?” and “Do you have chronic diseases?” Only one of them stayed in the model if a significant collinearity was identified.

We conducted multivariate linear regression analysis for examining the association between Internet use and outpatient clinic visits, including the confounding variables selected by linearity and collinearity checks. We regarded *P≤*.05 as statistically significant. We conducted all statistical analyses in this study using STATA/MP 11.0 (StataCorp LP) for Windows PC. This study was approved by the Research Ethics Committee of National Taiwan University Hospital (201510102W).

## Results

**Figure 1:**
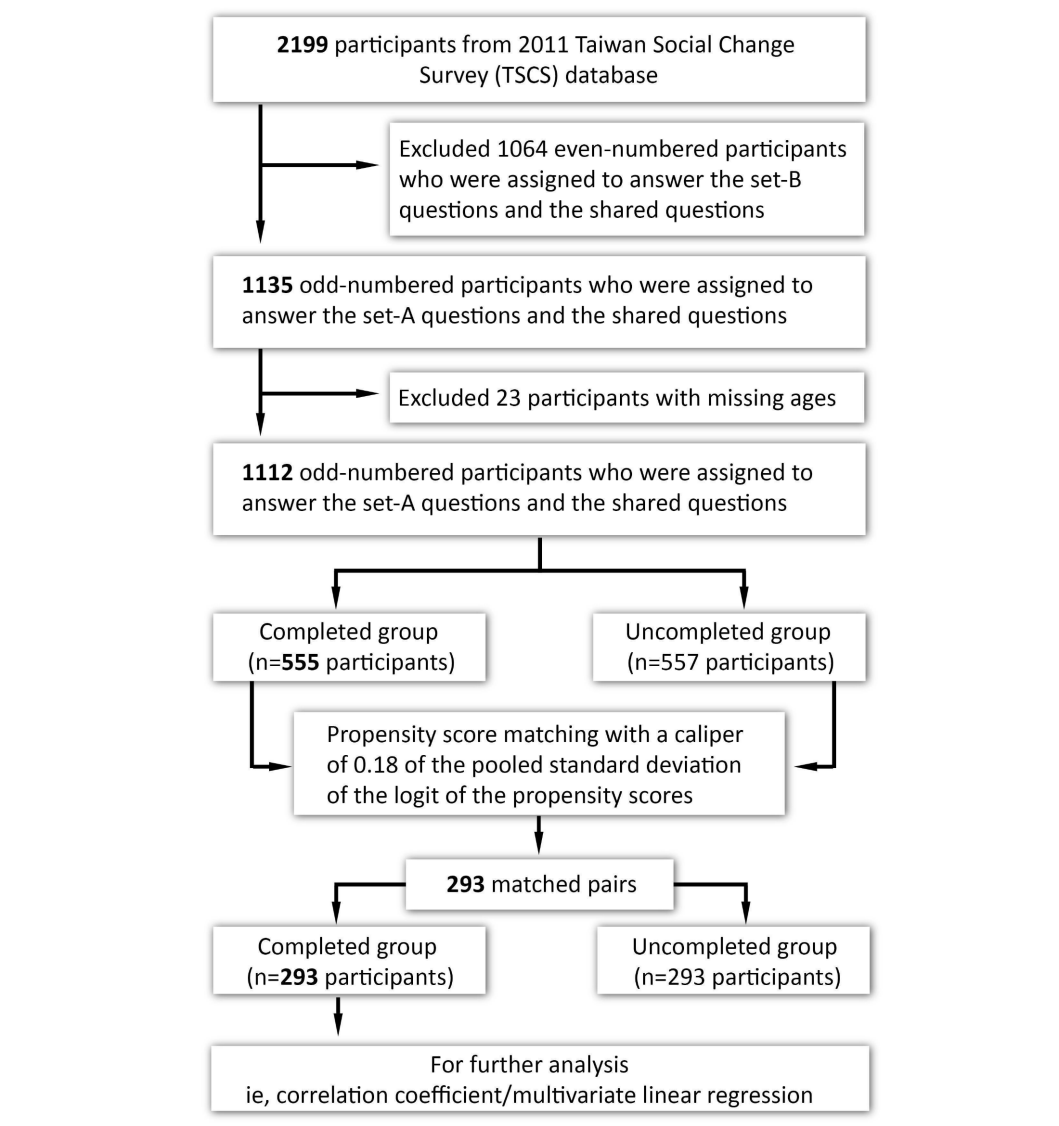
Participant selection.

A total of 2199 participants joined this study to answer the Health questionnaires. Among the 2199 Health questionnaires, 1064 (48.39%) were even numbered and assigned to answer the set-B questions and the shared questions. Given that set-A and set-B questions were established using different scales—that is, a scale from 1 to 5 was established for set-A questions but a scale from 1 to 6 was established for set-B questions—we thus excluded the participants who answered set-B questions from further analysis. Among the 1135 participants who were odd numbered and assigned to answer the set-A questions and the shared questions on the Health questionnaires, 23 (2.03%) did not provide their age and were thereby excluded. Therefore, we finally included 1112 Health questionnaires for propensity score matching using multivariate logistic regression (Figure 1).

A total of 1112 Health questionnaires were eligible for this study: 555 questionnaires did not have missing data (the completed group); 557 questionnaires (50.09%) had missing data (the uncompleted group). The participants in the completed group were significantly younger than those in the uncompleted group (*P*<.001). More male participants were in the completed group than in the uncompleted group with a borderline significance (*P*=.06). To avoid the potential risk of selection bias, we conducted propensity score matching to balance age and sex between the 555 questionnaires in the completed group and the 557 questionnaires in the uncompleted group (Table 1).

We established a propensity score model for matching a questionnaire answered by a participant in the completed group (n=555) to a questionnaire answered by a participant in the uncompleted group (n=557). The propensity score model for the completed group and uncompleted group included 2 independent variables: age and sex. It showed good discrimination (area under the receiver operating characteristic curve=0.83). We identified 293 matched pairs: 293 respondents from the completed group and 293 respondents from the uncompleted group. The mean (SD) propensity scores before matching were 0.67 (0.22) for the completed group and 0.33 (0.25) for the uncompleted group (*P*<.001). The mean (SD) propensity scores after matching were 0.55 (0.22) for the 293 participants from the completed and 0.51 (0.22) for the 293 participants from the uncompleted groups (*P*=.07). Age and sex were not significantly different between the 293 participants from the completed group and the 293 participants from the uncompleted group. The 293 participants from the completed group were eligible for bivariate analysis and multivariate linear regression analysis.

In examining the linear relationship between each independent variable and the number of outpatient clinic visits (Table 2), we found that participants who rated themselves healthier were negatively associated with making more outpatient clinic visits (*P*<.003). In comparison, people with chronic diseases, such as diabetes, hypertension, or hyperlipidemia, tended to have made more outpatient clinic visits (*P*<.001). The collinearity check using the Spearman rank correlation coefficient for “What is your health status?” and “Do you have chronic diseases?” showed a correlation coefficient of –.18 (*P*=.002). We therefore excluded “What is your health status?” from further analysis.

**Table 1:** Characteristics before and after propensity score matching for the completed group and uncompleted group.

Questionnaire item (score)		Before propensity score matching				After propensity score matching			
		Completed (n=555)	Uncompleted (n=557)	Missing data^a^	*P* value	Completed (n=293)	Uncompleted (n=293)	Missing data^a^	*P* value
		Mean (SD) or n (%)	Mean (SD) or n (%)			Mean (SD) or n (%)	Mean (SD) or n (%)		
**How often have you visited a doctor in the past 12 months? n (%)**				70	<.001			56	.47
	Never (1)	27 (4.86)	13 (2.76)			16 (5.46)	8 (3.38)		
	Once a year (2)	51 (9.19)	35 (7.19)			21 (7.17)	25 (10.55)		
	Several times a year (3)	383 (69.01)	295 (60.57)			205 (69.97)	160 (67.51)		
	Once a month (4)	78 (14.05)	118 (24.23)			42 (14.33)	34 (14.35)		
	Several times a month (5)	16 (2.88)	26 (5.34)			9 (3.07)	10 (4.22)		
How many hours do you use the Internet every day? mean (SD)									
		2.90 (2.69)	2.87 (3.15)	473	.93	2.28 (2.39)	2.95 (3.18)	212	.04
How many hours do you watch TV news every day? mean (SD)									
		0.98 (0.96)	1.10 (1.07)	3	.045	1.08 (1.11)	1.14 (1.05)	2	.49
How many years of school education did you receive? mean (SD)									
		14.23 (2.89)	9.66 (3.79)	71	<.001	13.79 (3.16)	10.78 (3.54)	7	<.001
Age in years, mean (SD)									
		37.21 (13.03)	57.91 (16.58)	0	<.001	43.89 (13.17)	45.94 (12.35)	0	.06
**Sex, n (%)**				0	.06			0	.16
	Female	265 (47.75)	297 (53.32)			163 (55.63)	146 (49.83)		
	Male	290 (52.25)	260 (46.68)			130 (44.37)	147 (50.17)		
**Residence, n (%)**				2	<.001			2	.03
	Rural	18 (3.24)	54 (9.73)			11 (3.75)	23 (7.90)		
	Urban	537 (96.76)	501 (90.27)			282 (96.25)	268 (92.10)		
**What is your marital status? n (%)**				2	<.001			2	.008
	Unmarried	245 (44.14)	59 (10.63)			77 (26.28)	57 (19.59)		
	Divorced	17 (3.06)	27 (4.86)			11 (3.75)	23 (7.90)		
	Widowed	2 (0.36)	88 (15.86)			2 (0.68)	10 (3.44)		
	Married	285 (51.35)	372 (67.03)			201 (68.60)	196 (67.35)		
	Other	6 (1.08)	9 (1.62)			2 (0.68)	5 (1.72)		
How many members, including you, are there in your family? mean (SD)									
		4.25 (1.86)	4.14 (2.13)	3	.35	4.20 (1.76)	4.23 (1.91)	3	.86
When you need help, your neighbors are willing to give you a hand. mean (SD)^b^									
		3.79 (0.89)	3.88 (0.84)	26	.09	3.84 (0.83)	3.82 (0.84)	21	.82
Are you satisfied with Taiwan’s health care system? mean (SD)^c^									
		3.46 (1.08)	3.62 (1.01)	13	.02	3.42 (1.12)	3.41 (1.05)	5	.91
Generally speaking, Taiwan’s physicians are trustworthy. mean (SD)^b^									
		3.56 (0.90)	3.49 (0.94)	9	.21	3.52 (0.90)	3.37 (1.01)	4	.07
**Do you smoke? n (%)**				2	.26			0	<.001
	No	454 (81.80)	439 (79.10)			248 (84.64)	210 (71.67)		
	Yes	101 (18.20)	116 (20.90)			45 (15.36)	83 (28.33)		

**Do you drink alcohol? n (%)**				3	<.001			0	.56
	No	272 (49.01)	345 (62.27)			162 (55.29)	155 (52.90)		
	Yes	283 (50.99)	209 (37.73)			131 (44.71)	138 (47.10)		
**Do you chew betel nut? n (%)**				0	<.001			0	<.001
	No	515 (92.79)	475 (85.28)			277 (94.54)	238 (81.23)		
	Yes	40 (7.21)	82 (14.72)			16 (5.46)	55 (18.77)		
**Did you receive a self-paid health checkup in the past 3 years? n (%)**				4	<.001			2	.30
	No	403 (72.61)	458 (82.82)			222 (75.77)	231 (79.38)		
	Yes	152 (27.39)	95 (17.18)			71 (24.23)	60 (20.62)		
What is your health status? mean (SD)^d^									
		2.63 (0.97)	2.44 (1.03)	2	<.002	2.61 (0.96)	2.58 (1.04)	0	.77
**Do you have chronic diseases? n (%)**				10	<.001			9	.25
	No	437 (78.74)	294 (53.75)			215 (73.38)	196 (69.01)		
	Yes	118 (21.26)	253 (46.25)			78 (26.62)	88 (30.99)		
**In the past 12 months, did you seek medical assistance from complementary and alternative medicine? n (%)**				61	.10			49	.61
	No	343 (61.80)	331 (66.73)			181 (61.77)	156 (63.93)		
	Yes	212 (38.20)	165 (33.27)			112 (38.23)	88 (36.07)		
**Are you satisfied with your quality of life? n (%)**				10	.92			3	.02
	Very dissatisfied or dissatisfied	115 (20.72)	112 (20.48)			51 (17.41)	73 (25.17)		
	Very satisfied or satisfied	440 (79.28)	435 (79.52)			242 (82.59)	217 (74.83)		
Is your household income higher than, lower than, or similar to other households in Taiwan? mean (SD)^e^									
		2.88 (0.63)	2.59 (0.75)	12	<.001	2.87 (0.64)	2.67 (0.71)	5	<.001

^a^The sample size of missing data in the uncompleted group next to itself for the item.

^b^Possible responses and their scores were strongly disagree (1), disagree (2), neutral (3), agree (4), and strongly agree (5).

^c^Possible responses and their scores were very dissatisfied (1), dissatisfied (2), neutral (3), satisfied (4), and very satisfied (5).

^d^Possible responses and their scores were bad (1), fair (2), good (3), very good (4), and excellent (5).

^e^Possible responses and their scores were much lower (1), lower (2), similar (3), higher (4), and much higher (5).

**Table 2:** Correlation coefficients between each independent variable and the number of outpatient clinic visits.

Variable	Correlation coefficient	*P* value
How many hours do you use the Internet every day?	.06	.27
How many hours do you watch TV every day?	.04	.48
How many years of school education did you receive?	–.10	.08
Age (1-year increment)	.11	.06
Sex (0=female, 1=male)	–.06	.34
Residence (0=rural, 1=urban)	–.01	.83
What is your marital status? (reference group: married)	–.04	.49
How many members, including you, are there in your family?	–.11	.06
When you need help, your neighbors are willing to give you a hand.	.02	.77
Are you satisfied with Taiwan’s health care system?	.01	.83
Generally speaking, Taiwan’s physicians are trustworthy.	.08	.15
Do you smoke? (0=no, 1=yes)	–.09	.11
Do you drink alcohol? (0=no, 1=yes)	–.07	.22
Do you chew betel nut? (0=no, 1=yes)	<.01	.99
Do you receive self-paid health checkup in the past 3 years? (0=No, 1=Yes)	.07	.22
What is your health status? (reference group: Bad)	–.18	.003
Do you have chronic diseases? (0=no, 1=yes)	.25	<.001
In the past 12 months, did you seek medical assistance from complementary and alternative medicine? (0=no, 1=yes)	.01	.83
Are you satisfied with your quality of life? (0=very dissatisfied/dissatisfied, 1=very satisfied/satisfied)	.06	.31
Is your household income higher than, lower than, or similar to other households in Taiwan?	.07	.24

After controlling for other confounding variables using multivariate linear regression for the 293 participants from the completed group, we found that Internet use was significantly associated with more outpatient clinic visits (*P*=.04) (Table 3). The adjusted *R*^2^ was .1195, indicating that 11.95% of the variance could be accounted for by this multivariate linear regression model.

**Table 3:** Multivariate linear regression on number of outpatient office visits (n=293)^a^.

Variable		Coefficient	*P* value
How many hours do you use the Internet every day?		0.04	.04
How many years of school education did you receive?		–0.03	.03
Age (1-year increment)		0.01	.20
Sex (0=female, 1=male)		–0.09	.32
**What is your marital status? (reference group: married)**			
	Unmarried	0.24	.08
	Divorced	0.15	.50
	Widowed	0.75	.15
	Other	–1.22	.02
How many members, including you, are there in your family?		–0.02	.50
Generally speaking, Taiwan’s physicians are trustworthy.		0.01	.76
Do you smoke? (0=no, 1=yes)		–0.19	.12
Do you drink alcohol? (0=no, 1=yes)		–0.11	.21
Did you receive a self-paid health checkup in the past 3 years? (0=no, 1=yes)		0.12	.22
Do you have chronic diseases? (0=no, 1=yes)		0.43	<.001
Is your household income higher than, lower than, or similar to other households in Taiwan?		0.04	.51

^a^The value of adjusted *R*^2^ for this multivariate linear regression model is .1195.

## Discussion

### Principal Findings

After controlling for age and sex using propensity core matching for the completed group and uncompleted group, and for other confounding variables using multivariate linear regression analysis, we found that spending more time using the Internet was significantly associated with making more outpatient clinic visits.

### Health-Related Information in the Media

Media such as newspapers, magazines, journals, television, and radio report not only information on daily life and their audiences’ interests, but also health-related information. One of the most important ways the media may help patients to correctly interpret health-related information is to present the information in an unbiased manner [19,20]. Nevertheless, this is not usually the case. Web-based health information may not be as correct as the information shown in textbooks or academic journals [21].

Diem et al [22] reported that, in television programs, the survival rates of people receiving cardiopulmonary resuscitation were significantly higher than the most optimistic survival rates reported in the literature. Chen et al [23] also reported that information reported in the major newspapers in Taiwan regarding the use of life supporting treatments for patients who are critically ill is too optimistic as indicated by the probability of survival. In addition to health-related information regarding life supporting treatments in the media, Moynihan et al [24] studied media coverage of the benefits and risks for three medications in leading national newspapers, local newspapers, and television networks in the United States. They concluded that media stories about medications included inadequate or insufficient information about the benefits, risks, and costs of the drugs.

One theme emerged from the above studies [22-24], which is that the media tends to show biased health-related information. Accordingly, the audience’s ability to analyze and evaluate the messages shown on a wide variety of media is critical for telling whether health-related information in the media is biased or unbiased.

### Internet Use and Outpatient Clinic Visits

The media plays an important role in raising awareness about health care services to patients and in shaping laypeople’s perceptions of and decision making about health care [25]. Our study found that laypeople who spend more time using the Internet are more likely to visit outpatient clinics. Several reasons may account for this phenomenon:

First, some studies reported that people who used the Internet to search for health-related information needed more help from health care professionals with interpreting and understanding the health-related information they obtained [11,26]. Another study reported that the controversial health-related information reported in the media might affect patients’ perceptions of and decision making about medical care [27]. In addition, several studies have shown that health-related information on the Internet is less likely to be accredited by the Health on the Net Foundation, and is therefore less reliable [28]. Such inconsistent quality may bring about significant anxiety for patients. As a result, patients who spend more time using the Internet may be more likely to visit outpatient clinics for clarification and interpretation of a kaleidoscope of health-related information. This, therefore, may increase physicians’ workloads, as they have to spend additional time discussing health-related information and reassuring patients [27].

Second, compared with nonseekers, adults seeking health-related information on the Web were more likely to rate themselves as having poor health [13]. Therefore, laypeople who spend more time using the Internet are more likely to visit outpatient clinics, not because of their Internet use, but because they tend to rate themselves as having poor health and, therefore, tend to seek professional advice about their health status.

Third, media literacy may account for our study result. Laypeople with better media literacy may not simply accept health-related information shown in the media. Instead, they may prefer to carefully digest health-related information obtained from the media by studying academic journals, seeking professional guidance by consulting health care professionals, and so on. Visiting outpatient clinics to obtain professional advice for health-related information shown in the media is the most convenient and least time-consuming way to get that guidance. As a result, people who frequently seek health-related information on the Web are significantly associated with making more outpatient clinic visits.

### Strengths and Limitations

We conducted this study, based on a sample derived from a random sample of the general population in Taiwan, to examine the relationship between Internet use and health-seeking behaviors as indicated by the number of outpatient clinic visits. We used sophisticated statistical methods to minimize the threat to external validity due to missing data and to control a large variety of confounding variables. In addition, the study results extended what is already known and from previously reported academic work by providing new data and by controlling for some confounding variables that were not controlled for (ie, the total number of family members and whether physicians are perceived as trustworthy) in previous studies [11-13]. Accordingly, we are confident that the results of this study are convincing and generalizable.

Nevertheless, there are some limitations in this study. This was a cross-sectional study using a questionnaire for the participants to recall the frequency of their Internet use and the frequency of their outpatient clinic visits. The causal relationship between Internet use and outpatient clinic visits is not as strong as in the design of a prospective cohort study. In addition, the frequency of Internet use recalled by the study respondents did not necessarily indicate that they had been searching for health-related information. This potential inaccuracy should be further considered when applying the results of this study.

Second, recall bias may also affect the outcome of this study. It could simply occur due to the differences in the accuracy or completeness of participants’ answers to the survey questions regarding their Internet use and outpatient clinic visits from the past year. Or, if participants were so sick that they had visited outpatient clinics several times, it would not have been easy for them to recall the frequency of their visits in the past year. Similarly, if participants only sometimes used the Internet, they might not have been able to recall the frequency of their Internet use.

Third, there may be concerns about the relationship between Internet use and the number of outpatient clinic visits, which was nonsignificant in univariate analysis (*P*=.27) but significant in multivariate analysis (*P*=.04). Several reasons may cause this phenomenon [29]: (1) interaction: we have checked the interaction between the time of using the Internet every day in hours and chronic diseases status, and identified that there is no interaction between these 2 variables (*P*=.57); (2) the effect of unbalanced sample size: the dataset for multivariate linear regression did not have unbalanced sample size; and (3) the influence of missing data: there were no missing data in the 293 questionnaires included in the multivariate linear regression model. There may be some other reasons associated with this “nonsignificant in univariate analysis but significant in multivariate analysis” phenomenon.

Fourth, we used propensity score matching to compare the subset of participants in the completed group with a subset of participants in the uncompleted group who were similar in age and sex [30-32]. The subsets of the completed and uncompleted groups selected using propensity score matching might not be well representative of their respective entire group. As a result, the generalizability of the study results might be of concern.

Fifth, the low adjusted *R*^2^ may be of concern. However, it is expected that the adjusted *R*^2^ value will be low in some fields. For example, fields that attempt to predict human behaviors typically have lower adjusted *R*^2^ values. Given that the nature of this study was to predict human behaviors, it is acceptable that our study results have an adjusted *R*^2^=.1195.

### Conclusions

The use of information technology, such as the Internet, to provide health-related information to the general population has grown extremely rapidly in the past decade and will continue to grow at a rapid pace in the future. Our study identified that Internet use is positively associated with frequency of outpatient clinic visits. The contradictory or diverse nature of Internet information might play an important role in the increasing frequency of outpatient clinic visits. In addition, patients’ ability to analyze and evaluate health-related information conveyed by a wide variety of media modes for telling whether this information is biased or unbiased may also influence their frequency of making outpatient clinic visits. Future studies may focus on how media literacy affects laypeople’s interpretation of health-related information and their health-seeking behaviors.

## Abbreviations

TSCS: Taiwan Social Change Survey
